# Do the respiration pulses induced by drying–rewetting matter for the soil–atmosphere carbon balance?

**DOI:** 10.1111/gcb.16163

**Published:** 2022-03-30

**Authors:** Johannes Rousk, Albert C. Brangarí

**Affiliations:** ^1^ Microbial Ecology Department of Biology Lund University Lund Sweden

**Keywords:** birch effect, carbon cycling, climate change, ecosystem drought, ecosystems, eddy covariance, soil microorganisms, soil moisture

## Abstract

We show that the explosive microbial and biogeochemical dynamics triggered by rewetting dry soil in laboratory experiments also has relevance in intact ecosystems. This highlights an opportunity to use predictions derived from laboratory studies to provide targets in ecosystem‐scale biogeochemical studies.

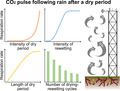

When life emerged from marine environments to colonize land surfaces, the new opportunities to thrive came at a cost: Suddenly, water was upgraded from a solvent for life chemistry and a transport medium to also become a critical resource of erratic supply. Since then, strategies to cope with drought have become an important adaptation determining the success of terrestrial organisms. Currently, the globe is undergoing rapid and pronounced climate change, subjecting terrestrial ecosystems to warmer conditions but also to more frequent and severe droughts (IPCC, 2021). Temperature and moisture are the strongest physical regulators of terrestrial ecosystem process rates, controlled by photosynthesizing plants and by decomposer microorganisms (Sierra et al., 2015). Thus, the functioning of whole ecosystems will respond to climate change, including plant nutrient provisioning along with the regulation of the balance of carbon (C) between atmosphere and soils, providing a feedback that could both accelerate and slow further climate change (Reichstein et al., 2013).

For nearly a century, a central problem in soil science has been the cascade of events triggered by the rewetting of dry soil by rainfall or irrigation. The reason for this careful and insistent study has been that rates of carbon dioxide (CO_2_) production and nutrient transformations as well as the size, composition and growth rates of the microbial community are surprisingly dynamic following the rewetting of dry soil—a phenomenon known as the “Birch effect” (Birch, 1958)—while the interlinkage of microbial structure and function has remained elusive to explain and predict. There is now an intensified need to understand ecosystem responses to climate change‐induced drought and thus the microbial control of the C cycle during drying and subsequent rewetting of soil. Motivated by this, a surge of research on microbial responses to drought and drying–rewetting events associated with cyclic ecosystem droughts has been and is being conducted. However, there is a cultural divide between (i) studies that target high time resolution assessments of microbial functional and structural responses and (ii) those that estimate biogeochemical process rates at the ecosystem scale.

While microbially targeted studies predominantly rely on small experimental units studied in laboratory conditions (microcosms) that often exclude plants, studies of ecosystem process rate normally are survey‐based, rely on large and rather expensive infrastructure, such as eddy covariance towers and associated analytical instruments, and for logistical reasons are difficult to replicate or expose to stringent and well‐controlled treatments (e.g., split‐plot designs). These divergent traditions have resulted in the contradiction of a self‐evident consensus within microbial ecology that drying–rewetting cycles are critical for soil–atmosphere C exchange while the consequences of the rewetting of dry soil for C cycling at ecosystem level remain mostly overlooked. Here, we will use predictions derived from microbially focused studies to target ecosystem C‐exchange datasets with conditions that should be conducive to pronounced respiration dynamics, to test if the microbial release of C from soil during drying–rewetting cycles contributes importantly to the soil–atmosphere C exchange in ecosystems and, therefore, if this should be a priority also in ecosystem scale biogeochemistry.

Controlled conditions in laboratory studies have allowed precise soil moisture treatments, facilitating the identification of microbial community processes and a comparison between samples collected from different ecosystems (Table S1), yielding many insights schematically reproduced in Figure 1a. For instance, we have learnt that the levels of moisture reached during drought and those during subsequent rewetting as well as the length and frequency of drying–rewetting events will affect the size of the CO_2_ released (Figure 1b), such that (1) the more complete the drying, the larger the CO_2_ pulse induced by rewetting, with a strong threshold moisture level occurring well below the plant wilting limit (e.g., Meisner et al., 2017); (2) rewetting to only a marginal moisture content, for instance from air‐dry to the wilting limit, will dampen the respiration pulse release, while the CO_2_ pulse released will increase with higher moisture levels all the way up to field capacity (e.g., Lado‐Monserrat et al., 2014); (3) long periods of drought preceding the rewetting will lead to more pronounced CO_2_ pulses that are also longer lasting (e.g., Tiemann & Billings, 2011); and (4) repeated cycles of drying–rewetting will incrementally reduce the size of the CO_2_ pulse (e.g., Miller et al., 2005).

**FIGURE 1:**
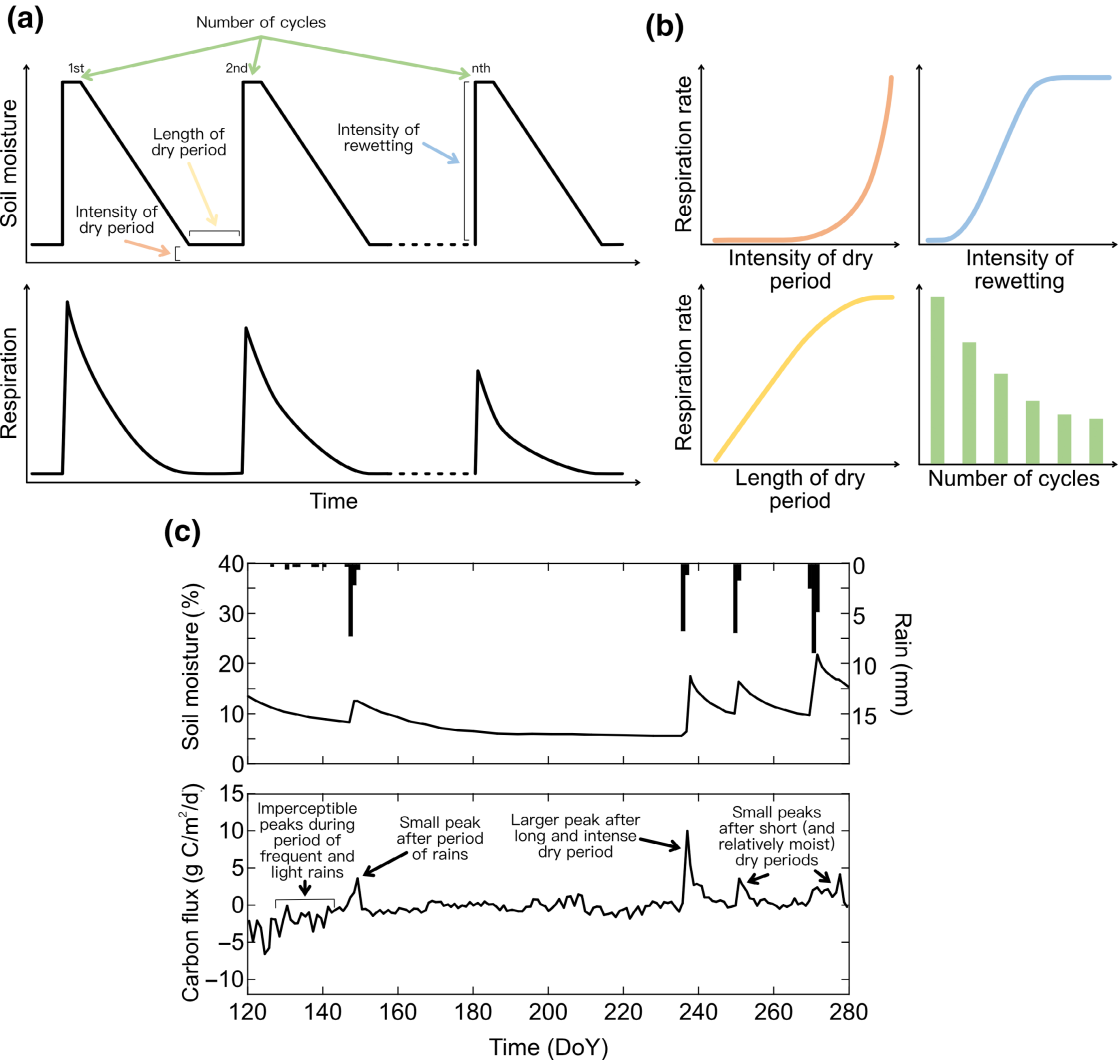
Conceptual figure showing the insights of soil–atmosphere C exchange at different scales. Panel (a): Conceptual representation of the influence of the patterns of drying–rewetting (upper box) on microbial respiration (lower box). Panel (b): Schematic representation of how the “intensity of dry period” (in orange), the “intensity of rewetting” (in blue), the “length of dry period” (in yellow), and the “number of cycles” of drying–rewetting (in green) affect the respiration rates, based on microbially targeted literature. Panel (c): Example of the ecosystem‐level C fluxes measured in an eddy covariance tower located in the Pianosa Island in Italy in 2003 (Inglima et al., 2009). Vertical bars represent daily precipitation (upper box) and the solid line shows changes in soil moisture (upper box), and daily net ecosystem exchange (lower box)

Equipped with these concrete predictions, we have explored published literature including data on CO_2_ exchange linked to variable soil moisture at ecosystem levels. Our targeted search has revealed that respiration pulses released during the microbial dynamics induced by drying–rewetting indeed *can* be sufficiently large to importantly characterize the soil–atmosphere C exchange (example in Figure 1c). While performing this survey, we noted that the definition of, for example, intensity and duration of natural weather events when not under experimental control is difficult and ambiguous. This renders the precise definition the driving parameters of the microbial responses to drying‐rewetting (“intensity of dry period,” “intensity of rewetting,” “length of dry period,” and “number of cycles”) elusive and challenging to standardize and use to compare results between studies. However, we did find clear examples that provide circumstantial evidence that is consistent with what microcosm studies of soil microbial responses in laboratory experiments have unearthed (Table S1). Specifically:

1. Large CO_2_ pulses appear when ecosystems experience rather dry conditions before rewetting, generally within or beyond the wilting point. This makes it more likely that pronounced respiration pulses are triggered by rewetting in ecosystems that are systematically exposed to droughts (e.g., Jia et al., 2014; Xu et al., 2004) but that they also are possible in generally moist ecosystems after periods of extreme drought (e.g., Feldman et al., 2021).
2. Larger CO_2_ pulses appear after more pronounced rewetting events. Very small rain events do not trigger a response that is observable at the ecosystem scale, but the occurrence of larger events does not guarantee large emissions unless they are preceded by a drought (e.g., Jarvis et al., 2007).
3. Longer dry periods prior to rewetting (i.e., lower frequencies of drying–rewetting) give rise to more pronounced CO_2_ pulses upon rewetting than do shorter dry periods (e.g., Inglima et al., 2009).

1. Successively smaller CO_2_ pulses after repeated rain events could not be directly observed. It is likely that this effect was masked by an interaction with other factors such as the intensity of drought between rainfall events (see points above).

The validation that microbially driven responses observed in controlled laboratory systems can also be detected at the ecosystem scale need to be used to guide our research priorities. These, and other insights from laboratory studies, warrant further targeted research where, for example, eddy covariance datasets are used to validate the patterns revealed in soil microcosm experiments. Priorities should include an assessment of:

- The influence of climate, including geographical gradients from humid to arid ecosystems, and also of experimental changes in precipitation within climates (e.g., rain‐out shelters).
- Land‐use differences, where changes in vegetation and disruption of soil structure with, for example, agricultural tillage, and fertilization regimes are likely to make the soil–atmosphere C balance more sensitive to rewetting after drought.
- The interaction between soil moisture and other factors associated with environmental change, including warming, nitrogen deposition, soil salinization, and other forms of industrial or urban pollution.

In addition, our observations also motivate the inclusion of new search images for ecosystem CO_2_‐exchange datasets. We postulate that observations of initial pulses of CO_2_ arising from soil after first rain after a drought have been excised from datasets in “noise‐cancelling” procedures, thus warranting reconsideration of the datasets available in data repositories (e.g., FLUXNET (*fluxnet*.*org*) or ICOS (*icos*‐*cp*.*eu*)).

We hope that our argument is heard, and that a better integration of the traditions of soil microbial ecology and the ecosystem scale flux measurements can be achieved, where hypotheses derived from controlled systems are tested in full‐scale ecosystems. This will create added value in both directions: (i) The relevance of soil microbial ecology at the ecosystem scale can be evaluated and reality checked, and (ii) the complex biogeochemical dynamics could be resolved into ecological mechanisms that can be better targeted in new experiments and also be incorporated into mechanistic models. Each one of these will be able to greatly advance the fields of microbial ecology and ecosystem‐scale biogeochemistry, respectively.

## Supporting information

Table S1
