# Supplementary material for: Do the respiration pulses induced by drying–rewetting matter for the soil–atmosphere carbon balance?
Source: Glob Chang Biol. 2022 Mar 30;28(11):3486–8. doi: 10.1111/gcb.16163 (PMC9314038; doi:10.1111/gcb.16163)
Supplement: Supplementary file 1 — Table S1 [file GCB-28-3486-s001.pdf]

Supporting information

Table S1: List of 10 examples of microbially-targeted laboratory experiments (top) and 10 examples of ecosystem-level observations (bottom) showcasing the heterogeneity of ecosystems where the Birch effect has been observed. Colored cells mark those studies that gathered/corroborated insights on how the intensity of dry period ("1"; in orange), the intensity of rewetting ("2"; in blue), the length of dry period ("3"; in yellow) or the number of cycles ("3"; in green) influenced the C emissions; the colour intensity depicts the degree of linkage between the study and the insight (brighter colors indicate qualitatively stronger links).

Study	Type of ecosystem	1	2	3	4
Microbially-targeted laboratory studies					
Barnard, R. L., Osborne, C. A., & Firestone, M. K. (2015). Changing precipitation pattern alters soil microbial community response to wet-up under a Mediterranean-type climate. *The ISME Journal 2015 9:4*, *9*(4), 946–957	Mediterranean grassland (USA). Precipitation pattern treatments				
de Nijs, E. A., Hicks, L. C., Leizeaga, A., Tietema, A., & Rousk, J. (2018). Soil microbial moisture dependences and responses to drying-rewetting: the legacy of 18 years drought. *Global Change Biology*, (July), 1–11	Continental heathland (Netherlands). Drought treatment				
Fierer, N., & Schimel, J. P. (2002). Effects of drying-rewetting frequency on soil carbon and nitrogen transformations. *Soil Biology and Biochemistry*, *34*(6), 777–787.	Mediterranean forest and grassland (USA). Soil moisture treatments				
Fischer, T. (2009). Substantial rewetting phenomena on soil respiration can be observed at low water availability. *Soil Biology and Biochemistry*, *41*(7), 1577–1579	Continental forests (Germany)				
Jones, A. R., Gupta, V. V. S. R., Buckley, S., Brackin, R., Schmidt, S., & Dalal, R. C. (2019). Drying and rewetting effects on organic matter mineralisation of contrasting soils after 36 years of storage. *Geoderma*, *342*(May 2018), 12–19	Dry subtropical pasture and grassland (Australia). Air-dry storage treatment				
Lado-Monserrat, L., Lull, C., Bautista, I., Lidón, A., & Herrera, R. (2014). Soil moisture increment as a controlling variable of the "Birch effect". Interactions with the pre-wetting soil moisture and litter addition. *Plant and Soil*, *379*(1–2), 21–34	Semiarid mediterranean forest (Spain). Litter amendment treatment				
Meisner, A., Leizeaga, A., Rousk, J., & Bååth, E. (2017). Partial drying accelerates bacterial growth recovery to rewetting. *Soil Biology and Biochemistry*, *112*, 269–276	Arctic soil (Greenland) and oceanic forest (UK)				
Miller, A. E., Schimel, J. P., Meixner, T., Sickman, J. O., & Melack, J. M. (2005). Episodic rewetting enhances carbon and nitrogen release from chaparral soils. *Soil Biology and Biochemistry*, *37*(12), 2195–2204	Mediterranean shrubland (USA). Litter amendment treatment				
Sawada, K., Funakawa, S., & Kosaki, T. (2016). Short-term respiration responses to drying-rewetting in soils from different climatic and land use conditions. *Applied Soil Ecology*, *103*, 13–21	Humidity gradient in forests, croplands, grasslands (Japan, Thailand, Kazakhstan)				
Tiemann, L. K., & Billings, S. A. (2011). Changes in variability of soil moisture alter microbial community C and N resource use. *Soil Biology and Biochemistry*, *43*(9), 1837–1847	Precipitation gradient in grasslands (USA)				
Ecosystem-scale studies					
Chi, J., Waldo, S., Pressley, S., O'Keeffe, P., Huggins, D., Stöckle, C., … Lamb, B. (2016). Assessing carbon and water dynamics of no-till and conventional tillage cropping systems in the inland Pacific Northwest US using the eddy covariance method. *Agricultural and Forest Meteorology*, *218–219*, 37–49	Mediterranean croplands (USA). Tillage practices				
Feldman, A. F., Chulakadabba, A., Short Gianotti, D. J., & Entekhabi, D. (2021). Landscape-Scale Plant Water Content and Carbon Flux Behavior Following Moisture Pulses: From Dryland to Mesic Environments. *Water Resources Research*, *57*(1), 1–20	Dry-humid continental gradient in grasslands, croplands, forests, shrublands (USA)				
Inglima, I., Alberti, G., Bertolini, T., Vaccari, F. P., Gioli, B., Miglietta, F., … Peressotti, A. (2009). Precipitation pulses enhance respiration of Mediterranean ecosystems: The balance between organic and inorganic components of increased soil CO2 efflux. *Global Change Biology*, *15*(5), 1289–1301	Mediterranean crop-pasture-shrubland (Italy)				
Jarvis, P., Rey, A., Petsikos, C., Wingate, L., Rayment, M., Pereira, J., … Valentini, R. (2007). Drying and wetting of Mediterranean soils stimulates decomposition and carbon dioxide emission: The "Birch effect." *Tree Physiology*, *27*(7), 929–940	Mediterranean and semiarid ecosystems (Italy and Portugal)				
Jia, X., Zha, T. S., Wu, B., Zhang, Y. Q., Gong, J. N., Qin, S. G., … Peltola, H. (2014). Biophysical controls on net ecosystem CO2exchange over a semiarid shrubland in northwest China. *Biogeosciences*, *11*(17), 4679–4693	Semiarid continental shrubland (China)				
Tang, J., Misson, L., Gershenson, A., Cheng, W., & Goldstein, A. H. (2005). Continuous measurements of soil respiration with and without roots in a ponderosa pine plantation in the Sierra Nevada Mountains. *Agricultural and Forest Meteorology*, *132*(3–4), 212–227	Mediterranean forest (USA)				
Unger, S., Máguas, C., Pereira, J. S., David, T. S., & Werner, C. (2010). The influence of precipitation pulses on soil respiration - Assessing the " Birch effect" by stable carbon isotopes. *Soil Biology and Biochemistry*, *42*(10), 1800–1810	Semiarid mediterranean forest (Portugal)				
Williams, C. A., Hanan, N., Scholes, R. J., & Kutsch, W. (2009). Complexity in water and carbon dioxide fluxes following rain pulses in an African savanna. *Oecologia*, *161*(3), 469–480	Semiarid savannah forest (South Africa)				
Xu, L., Baldocchi, D. D., & Tang, J. (2004). How soil moisture, rain pulses, and growth alter the response of ecosystem respiration to temperature. *Global Biogeochemical Cycles*, *18*(4), 1–10	Mediterranean grassland and savannah (USA)				
Zhou, Y., Li, X., Gao, Y., He, M., Wang, M., Wang, Y., … Li, Y. (2020). Carbon fluxes response of an artificial sand-binding vegetation system to rainfall variation during the growing season in the Tengger Desert. *Journal of Environmental Management*, *266*	Continental and desert shrubland (China)				
